# Comprehensive Evaluation of Triptolide’s Therapeutic Mechanisms in Diabetic Kidney Disease *via* Meta-Analysis, Network Pharmacology, Molecular Docking, and Mendelian Randomization

**DOI:** 10.2174/0113816128367671250714100708

**Published:** 2025-07-23

**Authors:** Jing Ni, Siyuan Song, Yi Wei, Qiling Zhang, Wei Li, Jiangyi Yu

**Affiliations:** 1 Department of Endocrinology, Jiangning District of Nanjing Chinese Medicine Hospital, Affiliated Jiangning Hospital of Chinese Medicine, China Pharmaceutical University, Nanjing, China;; 2 Nanjing University of Chinese Medicine, Nanjing, China;; 3 Department of Endocrinology, Jiangsu Provincial Hospital of Chinese Medicine, Affiliated Hospital of Nanjing University of Chinese Medicine, Nanjing, China;; 4 Department of Neurology, Jiangning District of Nanjing Chinese Medicine Hospital, Affiliated Jiangning Hospital of Chinese Medicine, China Pharmaceutical University, Nanjing, China

**Keywords:** Triptolide, diabetic kidney disease, meta-analysis, network pharmacology, molecular docking, mendelian randomization, immunomodulation, inflammation

## Abstract

**Introduction:**

Diabetic kidney disease (DKD) is a devastating complication of diabetes for which there are few potent treatments.Triptolide (TP), an active compound from *Tripterygium wilfordii*, has shown potential in early studies, but its therapeutic mechanisms in DKD are not fully understood. This study aims to systematically evaluate TP’s efficacy and mechanisms using meta-analysis, network pharmacology, molecular docking, and Mendelian randomization (MR).

**Methods:**

A comprehensive search across Chinese and English databases identified animal randomized controlled trials (RCTs) assessing the effects of TP on DKD. A total of 27 studies were incorporated, and a meta-analysis was conducted *via* Review Manager. TP's drug and disease targets were identified through network pharmacology and molecular docking, while bioinformatics methods were employed to explore the mechanisms. MR analysis was performed to assess potential causal relationships between TP and DKD-related targets.

**Results:**

Meta-analysis showed that TP significantly reduced urinary protein, blood lipids, and glucose levels, while improving renal function, renal weight, and renal index (all *p* < 0.05). Seven core targets—IFNG, CXCL8, TNF, TGFB1, IL2, IL4, and RELA—were identified *via* network pharmacology, involving key pathways such as lipid-atherosclerosis, AGE-RAGE, and IL-17 signaling. Molecular docking demonstrated strong binding affinities between TP and these targets, with binding energies below -7.00 kJ/mol. Although MR analysis did not establish direct causal relationships between these core genes and DKD, a significant negative correlation between TNF, IL4, and GFR was observed, suggesting their involvement in DKD progression.

**Discussion:**

TP may exert therapeutic effects on DKD through coordinated regulation of immune and inflammatory pathways. The integration of multi-omics approaches supports its multi-target pharmacological mechanisms. Although MR analysis did not confirm direct causal relationships, the identified gene associations further reinforce the potential biological relevance of TP. However, this study was primarily based on public datasets and lacks experimental validation *in vivo* and *in vitro*.

**Conclusion:**

TP exerts therapeutic effects on DKD through multi-target and multi-pathway mechanisms, primarily involving immunomodulation, anti-inflammation, anti-oxidation, and anti-fibrosis processes.

## 1 INTRODUCTION

Diabetic kidney disease (DKD) is kidney damage resulting from prolonged hyperglycemia and represents one of the major microvascular complications of diabetes. Clinically, DKD is characterized by microalbuminuria, macroalbuminuria, and edema, along with a gradual deterioration in renal function, making it the leading cause of end-stage renal disease (ESRD) [1]. The presence of DKD significantly increases the risk of adverse cardiovascular and renal events, especially when the estimated glomerular filtration rate (eGFR) falls below 60 mL/min/1.73 m^2^, at which point cardiovascular and renal risks escalate. Similarly, the urinary albumin-to-creatinine ratio (UACR) exhibits a linear relationship with the risk of cardiovascular and renal outcomes [1, 2]. As one of the primary drivers of diabetes-related mortality, DKD has imposed an increasing economic burden on diabetes management worldwide [3].

Current therapeutic approaches focus on managing glycemia, blood pressure, and lipid levels. Renin-angiotensin system inhibitors (RASi), sodium-glucose cotransporter 2 inhibitors (SGLT2i), and novel mineralocorticoid receptor antagonists (MRA) have been shown to reduce proteinuria [4]. However, these drugs are still considered suboptimal, contributing to a higher financial burden and potentially leading to severe side effects such as diabetic ketoacidosis [5], acute kidney injury [6], and severe hyperkalemia [7]. Consequently, the incidence of DKD and its progression to ESRD continue to rise [1-3].

Triptolide (TP), an active component extracted from the traditional Chinese medicinal herb *Tripterygium wilfordii*, has demonstrated potent anti-inflammatory, immunomodulatory, anti-tumor, and antifibrotic activities [8-10]. Several animal studies have shown TP's renal protective effects in DKD, including the protection of podocytes in diabetic mice by inhibiting hyperglycemia-induced oxidative stress and inflammatory responses [11, 12]. Additionally, TP has been found to suppress podocyte epithelial-mesenchymal transition by inhibiting the activation of the Wnt3α/ β-catenin or TGF-β/Smad signaling pathways, thereby exerting its renal protective function [13, 14]. Nonetheless, systematic reviews and bioinformatics-based evaluations of TP's effectiveness are limited.

Meta-analysis, a statistical approach, systematically combines results from multiple studies, enhancing statistical power and providing more reliable conclusions [15]. Network pharmacology, a novel research method grounded in systems biology and multi-faceted network analysis, aims to uncover the mechanisms of drug action through multiple targets and pathways. Molecular docking, a computational technique, predicts interactions between small molecules and protein targets [16].

Mendelian randomization (MR) utilizes single nucleotide polymorphisms (SNPs) identified in genome-wide association studies (GWAS) as instrumental variables (IVs) to assess causal relationships between exposure factors and disease outcomes [17]. Building on this foundation, the present study aims to explore the potential mechanisms of TP in treating DKD using multidimensional databases, including meta-analysis, network pharmacology, molecular docking, and MR analysis.

## 2 METHODS

Given that no animal or human experimentation was conducted in this study, ethical clearance and participant consent were not required.

## 3 META-ANALYSIS

### 3.1 Literature Search Strategy

The search included the following Chinese databases: China National Knowledge Infrastructure (CNKI), VIP Journal Service (VIP), Wanfang, and China Biology Medicine Database (CBM). In addition, English databases searched included PubMed, Web of Science, Cochrane Library, and Embase. The search covered publications up to January 2025. Literature was retrieved using subject headings and free-text terms, including “Triptolide”, “Diabetic Kidney Disease”, “Diabetic Nephropathies” and animal models like “Mus”, “Mouse”, “Mice” and “Rat” in both languages (see S1_Data for the detailed PubMed search strategy).

### 3.2 Inclusion and Exclusion Criteria

#### 3.2.1 Inclusion Criteria

1) Study Type: Published randomized controlled trials (RCTs) on animals written in either English or Chinese. 2) Subjects: Mice successfully modeled for DKD. 3) Interventions: The treatment group received Triptolide, while the control group was administered an equal volume of inactive liquid (such as saline, distilled water, or PBS buffer), a blank control, or standard treatments based on clinical guidelines, including angiotensin-converting enzyme inhibitors (ACEIs) or angiotensin II receptor blockers (ARBs).

#### 3.2.2 Exclusion Criteria

1) Non-animal studies (*e.g*., *in vitro* cell experiments, clinical trials). 2) Non-experimental literature (*e.g*., reviews, conference papers, research achievements, or meta-analyses). 3) Studies involving confounding factors (*e.g*., combined use of other herbal ingredients or non-DKD subjects). 4) Use of additional hypoglycemic drugs during the treatment period. 5) Duplicate experimental data. 6) Inability to extract experimental data. 7) Unavailability of full-text articles.

### 3.3 Outcome Measures

Primary outcomes included the renal index (KI), renal weight (KW), renal function markers (including serum creatinine (SCr) and blood urea nitrogen (BUN)), proteinuria (24-hour urinary protein (24 h UTP), urinary albumin (24 h UAL), and urinary albumin-to-creatinine ratio (UACR)), lipid profile (total cholesterol (TC), triglycerides (TG)), and fasting blood glucose or random blood glucose.

### 3.4 Data Extraction and Quality Assessment

Using the search strategy mentioned above, literature was retrieved and screened according to the inclusion and exclusion criteria. Baseline data from the eligible studies were independently extracted by two researchers, covering details such as the author, publication year, animal model (species, sex, age, weight, sample size, modeling method, and criteria for successful modeling), interventions (drug type, dosage, and treatment duration), and outcome measures. Any disagreements were settled through discussion or by consulting third-party experts. The quality of the included studies was evaluated using SYRCLE's risk of bias tool for animal studies [18], with the results categorized as low risk, high risk, or unclear risk.

### 3.5 Statistical Analysis

Meta-analysis was conducted using Review Manager 5.4. Heterogeneity was assessed using Chi-square and *I*^2^ tests, with a Fixed Effects Model (FEM) applied when *P* ≥ 0.1 and *I*^2^ ≤ 50%, and a Random Effects Model (REM) otherwise. Standardized mean differences (SMD) with 95% confidence intervals (CI) were computed for continuous variables, with statistical significance defined as *P* < 0.05. Subgroup analyses were performed based on triptolide dosage: micro-dose (TP ≤ 100 µg/(kg·d)), low dose (100 < TP ≤ 200 µg/(kg·d)), medium dose (200 < TP ≤ 400 µg/(kg·d)), and high dose (TP > 400 µg/(kg·d)). The results were presented in forest plots and tables. Sensitivity analysis was performed using the leave-one-out method, and publication bias was assessed using a funnel plot.

### 3.6 Network Pharmacology

#### 3.6.1 Drug and Disease Target Screening

The targets of Triptolide (TP) were determined through the TCMSP database (https://tcmsp-e.com/tcmsp.php), and human-derived targets were filtered and standardized through the UniProt database (https://www.uniprot.org/), resulting in gene names and Swiss-Prot IDs. These were used to construct a TP-related target database. For disease targets, the keyword “Diabetic Nephropathy” was used to search the GeneCards (https://previous.genecards.org/), OMIM (https://omim.org/), and TTD (https://db.idrblab.net/ttd/) databases. Targets with a relevance score ≥5 in GeneCards were retained, and targets from the three databases were merged and deduplicated to create a DKD target database (see S3_Data).

### 3.7 PPI Network and Core Target Identification

The potential targets of TP for DKD were identified by finding the overlap between drug and disease targets using the Venny 2.1.0 online tool (https://bioinfogp.cnb.csic.es/tools/venny/). The potential target gene names were then entered into the STRING database (https://cn.string-db.org/) with the species set to “Homo sapiens” for protein-protein Interaction (PPI) analysis, using the default minimum interaction threshold of 0.4. The resulting PPI network was exported as a TSV file and imported into Cytoscape 3.10.0 for visualization, with node size reflecting the degree of interaction. Topological analysis was performed using the Centiscape 2.2 plugin in Cytoscape, and core targets were identified based on the average values of degree centrality, closeness centrality, and betweenness centrality.

### 3.8 GO and KEGG Enrichment Analysis

The potential targets identified were imported into the DAVID database (https://david.ncifcrf.gov/) for Gene Ontology (GO) and Kyoto Encyclopedia of Genes and Genomes (KEGG) pathway enrichment analysis, with the species limited to “Homo sapiens” and results filtered based on *P* <0.05. GO enrichment analysis covered three categories: biological processes (BP), cellular components (CC), and molecular functions (MF). For each category, the top 10 terms were selected based on *P*-value rankings. The KEGG pathway analysis extracted the top 20 terms, which were visualized using the Bioinformatics online platform (https://www.bioinformatics.com.cn/).

### 3.9 Construction of the Network

Based on the TP-related target database and the top 20 KEGG pathways, .work and .type files were created and imported into Cytoscape 3.10.0 to construct a TP-target-DKD-signaling pathway interaction network.

### 3.10 Molecular Docking

The 3D structure of TP was obtained from the TCMSP database, and the Swiss-Prot IDs of the core targets were entered into the Protein Data Bank (PDB) (https://www.rcsb.org/) to retrieve the corresponding receptor protein PDB files. PyMOL was used to prepare the target proteins by removing water and ligands. The structures of TP and the target proteins were further processed using AutoDock Tools 1.5.7 and saved as ligand.pdbqt and protein.pdbqt files, respectively. Molecular docking was carried out using AutoDock Vina to identify the conformation with the lowest binding energy. The results were visualized using PyMOL.

### 3.11 MR Analysis

#### 3.11.1 Data Sources

In this study, the core genes were used as exposure factors, while diabetic kidney disease (DKD), glomerular filtration rate (GFR), and urinary albumin-to-creatinine ratio (UACR) were used as outcome factors. The corresponding GWAS data for both exposure and outcome variables were obtained from the Open GWAS database (https://gwas.mrcieu.ac.uk/), the GWAS Catalog database (https://www.ebi.ac.uk/gwas/home), and the CKD Gen database (http://ckdgen.imbi.uni-freiburg.de/), respectively.

### 3.12 Instrumental Variable Selection

SNPs that were highly correlated with the exposure factors but had no direct relationship with the outcome variables were selected as instrumental variables. The selection criteria were as follows: 1. Independent SNPs associated with the core genes were extracted from the GWAS data, with a threshold of *P* < 5 × 10^-8^. If additional instrumental variables were needed, a threshold of *P* < 5 × 10^−6^ was applied; 2. To remove linkage disequilibrium, parameters of r^2^ = 0.001 and kb = 10,000 were used; 3. To avoid weak instrument bias, the F-statistic for each set of exposure instruments was calculated according to the method described by Lv *et al*. [19]. SNPs with F<10 were considered weak instruments and removed.

### 3.13 Two-sample MR Analysis

Three MR analysis methods were applied to assess the causal relationship between exposure factors and outcome variables, with the inverse variance weighted (IVW) method being the primary approach. The MR-Egger regression and the weighted median (WM) methods were used as supplementary analyses.

### 3.14 Pleiotropy and Heterogeneity Assessment

Pleiotropy was assessed using the MR-Egger regression intercept test, and if *P* > 0.05, it was considered that no pleiotropic bias existed. Heterogeneity was evaluated using Cochran’s Q test, and if *P* < 0.05, heterogeneity was indicated.

### 3.15 Sensitivity Analysis

Sensitivity analysis was performed using the leave-one-out method, where each instrumental variable was sequentially removed to recalculate the combined effect size and assess the stability of the results.

### 3.16 Statistical Software

The MR analyses were performed using R (version 4.4.0) along with the TwoSampleMR and MR-PRESSO packages for statistical analysis.

## 4 RESULTS

### 4.1 Meta-analysis Results

#### 4.1.1 Literature Search and Inclusion Results

Following the literature search strategy described above, a total of 238 relevant studies were initially identified. Studies marked as “retracted” or “alerted” were excluded during the search. After removing duplicates, 122 studies remained. Further screening based on titles, abstracts, and full texts resulted in the inclusion of 27 studies.

### 4.2 Characteristics of Included Studies

The 27 included studies consisted of 6 English-language studies and 21 Chinese-language studies. All studies evaluated the effects of TP on DKD, with 7 studies comparing TP with ARB/ACEI treatments for DKD. Among these studies, 21 used male Sprague-Dawley (SD) or Wistar rats, and 6 used transgenic or mutant mice (Table **1**) [20-44].

### 4.3 Risk of bias Assessment for Included Studies

**Fig. (1):**
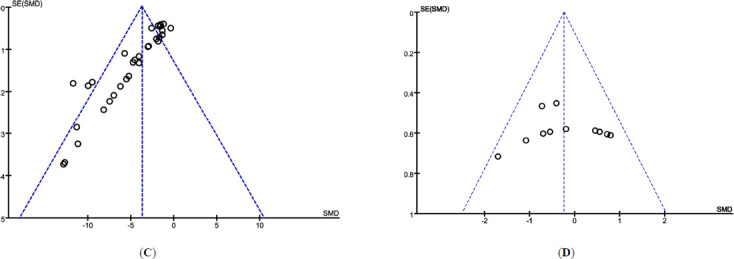
Results of Meta-analysis. (**A**) Risk of bias assessment in the included studies. (**B**) Subgroup analysis of the effect of different doses of TP on 24 h UAL in DKD. (**C**) Publication bias analysis of TP’s effect on proteinuria. (**D**) Comparison of TP and ARB/ACEI drugs on proteinuria.

The SYRCLE risk of bias tool for animal studies was applied to evaluate the 27 included studies. The results were classified into three categories: “Low risk,” “Unclear risk,” and “High risk.” All studies mentioned random group allocation, and five studies employed random number tables, resulting in a “Low risk” rating. Two studies [24, 29] employed weight-based random allocation, and these were classified as “High risk.” The remaining studies did not specify randomization methods and were rated as “Unclear risk.” Four studies [24, 28, 40, 41] explicitly reported baseline characteristics of the animals and were rated as “Low risk,” while the rest were rated as “Unclear risk” due to a lack of information. None of the studies reported allocation concealment, resulting in an “Unclear risk” rating. Similarly, none of the studies described whether the animals were randomly housed or if blinding was applied to researchers, also resulting in “Unclear risk” ratings. Three studies [21, 30, 40] mentioned “random” or “arbitrary” animal selection without specifying methods, leading to an “Unclear risk” rating for measurement bias, while the rest were rated as “Low risk.” Five studies [28, 29, 36, 39, 41] had incomplete outcome data, resulting in “Unclear risk” ratings for attrition bias. Reporting bias was deemed “Low risk” across all studies, except for one study [37] that suggested potential other biases and was rated as “High risk.” For the rest, bias risk was unknown and thus rated as “Unclear risk” (Fig. **1A**).

### 4.4 Evaluation of the Efficacy in Reducing Renal Weight

1) Analysis of KW: Five studies were included, and heterogeneity analysis indicated *I*^2^ = 78%, *p* < 0.00001, so a Random Effects Model (REM) was applied. The findings indicated that TP significantly reduced renal weight in DKD rats (SMD = −0.71, 95% CI [−1.41, −0.0^2^], *P* = 0.04). 2) Analysis of KI index: Thirteen studies were included, and heterogeneity analysis indicated *I*^2^ = 86%, *p* < 0.00001, so an REM was used. The findings demonstrated that TP significantly reduced the renal index in DKD rats (SMD = −3.61, 95% CI [−4.50, −2.^72^], *p* < 0.00001) (Table **2**).

### 4.5 Evaluation of the Efficacy in Reducing Proteinuria

The meta-analysis revealed that TP had a significant effect on reducing proteinuria. 1) Analysis of 24 h UAL (Fig. **1B** and Table **2**): Thirteen studies were included, and heterogeneity analysis indicated *I*^2^ = 82%, *p* < 0.00001, prompting the use of a Random effects Model (REM). Subgroup analyses were performed based on triptolide dosage: micro-dose (TP ≤ 100 µg/(kg·d)), low dose (100 < TP ≤ 200 µg/(kg·d)), medium dose (200 < TP ≤ 400 µg/(kg·d)), and high dose (TP > 400 µg/(kg·d)). The results indicated that TP at various dosages significantly reduced 24 h UAL in DKD rats, with the optimal effect observed at a dosage of 200 < TP ≤ 400 µg/(kg·d). Meanwhile, subgroup analyses were conducted based on different diabetes induction methods. The results showed that TP significantly reduced 24 h UAL in both the STZ-only induced model (SMD = −5.00, 95% CI [−6.44, -3.^55^]) and the HFD + STZ induced model (SMD = −2.59, 95% CI [−3.72, -1.^46^]) (*p* < 0.001). These findings suggest that the nephroprotective effect of TP may be independent of the type of diabetes (type 1 or type 2 diabetes mellitus) (Fig. **S1**.). 2) Analysis of 24 h UTP (Table **2**): Four studies were included, and heterogeneity analysis showed *I*^2^ = 0, *P* = 1.00, thus, a Fixed Effects Model (FEM) was applied. Meta-analysis results showed that TP significantly reduced 24 h UTP in DKD rats (SMD = −3.12, 95% CI [−3.75, −2.^49^], *P* < 0.00001). 3) Analysis of UACR (Table **2**): Nine studies were included, and heterogeneity analysis indicated *I*^2^ = 94%, *P* < 0.00001, so a REM was applied. Meta-analysis results showed that TP significantly reduced UACR in DKD rats (SMD = −4.73, 95% CI [−6.70, −2.^76^], *P* < 0.00001).

### 4.6 Evaluation of the efficacy in Improving Renal Function

The meta-analysis results indicated that TP treatment significantly improved renal function in DKD rats. 1. For SCr: Nineteen studies were included. The heterogeneity test showed *I*^2^ = 79%, *P* < 0.00001, and the REM was applied. The results showed that TP significantly reduced SCr (SMD = −0.89, 95% CI [−1.34, −0.^44^], *P* = 0.0001). 2. For BUN: Thirteen studies were included. The heterogeneity test showed *I*^2^ = 78%, *P* < 0.00001, and the REM was applied. TP treatment significantly reduced BUN (SMD = −0.96, 95% CI [−1.43, −0.^48^], *P* < 0.0001). All these results were statistically significant (Table **2**). To minimize the impact of heterogeneity on the results, subgroup analyses were conducted based on TP dosage. The findings revealed that when TP ≤200 µg/(kg·d), a significant protective effect on SCr was observed (*P* < 0.001). However, further increases in TP dosage did not yield a statistically significant difference compared to the DKD control group (Fig. **S2**). When TP dosage ranged from 100 µg/(kg·d) to 400 µg/(kg·d), subgroup analysis indicated a protective effect on BUN (*P* < 0.001). In contrast, no significant difference was observed between the DKD control group and the groups with TP ≤100 µg/(kg·d) or TP > 400 µg/(kg·d) (Fig. **S3**).

### 4.7 Evaluation of the efficacy in Regulating Blood Lipids

The meta-analysis results demonstrated that TP treatment significantly improved lipid profiles in DKD rats. 1. For TC: Fifteen studies were included. The heterogeneity test showed *I*^2^ = 71%, *P* < 0.00001, and an REM was used. TP treatment significantly lowered TC levels (SMD = −1.33, 95% CI [−1.73, −0.93], *P* < 0.00001). 2. For TG: Fifteen studies were included. The heterogeneity test showed *I*^2^ = 77%, *P* < 0.00001, and the REM was used. TP led to a significant reduction in TG levels (SMD = −1.47, 95% CI [−1.92, −1.0^1^], *P* < 0.00001). All of these results were statistically significant (Table **2**).

### 4.8 Evaluation of the Impact on Blood Glucose

The meta-analysis results showed that TP treatment significantly improved blood glucose levels in DKD rats. A total of 22 studies were included, and the heterogeneity analysis indicated *I*^2^ = 75%, *P* < 0.00001. The REM was applied, and TP significantly reduced blood glucose (SMD = −0.55, 95% CI [−0.87, −0.^22^], *P* = 0.001), with a statistically significant difference (Table **2**).

### 4.9 Evaluation of TP *vs*. ARB/ACEI in Improving Proteinuria

The meta-analysis results revealed that TP was superior to ARB/ACEI drugs in improving 24 h UTP. The detailed results are as follows: 1) For 24 h UAL: Four studies were included, and the heterogeneity test showed *I*^2^ = 40%, *P* = 0.09. Using an REM, the results showed that TP had no statistically significant difference compared to ARB/ACEI drugs (SMD = −0.24, 95% CI [−0.68, −0.^20^], *P* = 0.28). 2) For 24 h UTP: One study was included, and a FEM was applied. The results demonstrated that TP significantly reduced 24 h UTP compared to ARB/ACEI treatments (SMD = −1.17, 95% CI [−1.85, −0.^48^], *P* = 0.00008). 3) For UACR: Two studies were included, and the heterogeneity analysis showed *I*^2^ = 0, *P* = 0.65, so an REM was applied. The results showed no statistically significant difference between TP and ARB/ACEI drugs (SMD = −0.24, 95% CI [−0.50, 0.0^1^], *P* = 0.06). In summary, TP had a statistically significant effect on reducing 24 h UTP, but there was no significant difference compared to ARB/ACEI drugs in 24 h UAL and UACR. Due to the limited number of studies included, the reliability of the results is low (Table **3**).

### 4.10 Evaluation of TP *vs.* ARB/ACEI in Improving Renal Function

The meta-analysis results indicated that there was no statistically significant difference between TP and ARB/ACEI treatments in enhancing renal function. 1. For SCr: Four studies were included, and the heterogeneity test showed *I*^2^ = 0, *P* = 0.55. Using a FEM, the results showed no significant difference between TP and ARB/ACEI drugs (SMD = −0.21, 95% CI [−0.67, 0.^25^], *P* = 0.37). 2. For BUN: Three studies were included, and the heterogeneity test showed *I*^2^ = 0, *P* = 0.65. A FEM was applied, and the results showed no statistically significant difference between TP and ARB/ACEI drugs (SMD = −0.50, 95% CI [−1.01, 0.00], *P* = 0.05) (Table **3**).

### 4.11 Sensitivity Analysis

Sensitivity analyses were performed on outcomes including KI, KW, 24 h UAL, 24 h UTP, UACR, SCr, BUN, TC, TG, and fasting/random blood glucose by sequentially excluding individual studies. Only KW was identified as having one study that was a major source of heterogeneity [38]. After excluding this study, the meta-analysis results showed an SMD of −0.37 (95% CI [−0.90, 0.^16^], *P* = 0.17), and the difference was not statistically significant. For all other outcomes, the sensitivity analysis results were not significantly different from the original meta-analysis, indicating that the results for these indicators, except for KW, are relatively stable and reliable.

### 4.12 Publication Bias Analysis

Publication bias for TP treatment of DKD was assessed using 24 h UAL as the outcome measure, and an inverted funnel plot was generated. The plot showed obvious asymmetry around the central line, suggesting the presence of publication bias (Fig. **1C**). This bias may result from the high number of positive results in the included studies or the lack of publication of negative findings. When assessing publication bias for TP *versus* ARB/ACEI treatment of DKD using 24 h UAL as the outcome, the inverted funnel plot appeared symmetrical, indicating no significant publication bias (Fig. **1D**).

### 4.13 Network Pharmacology Results

#### 4.13.1 Establishment of a Potential Target Database

**Fig. (2):**
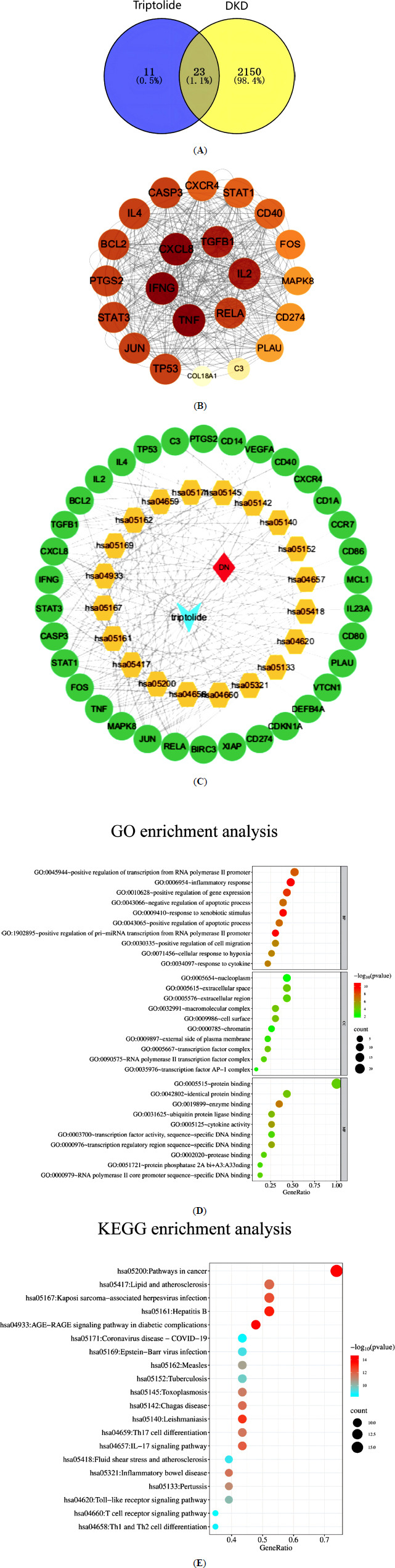
Network pharmacology analysis of TP in the treatment of DKD. (**A**) Acquisition of intersection target; (**B**) Intersection target protein-protein interaction network diagram; (**C**) Network diagram of TP- target- DKD -signaling pathway; (**D** and **E**) Bubble diagrams for GO and KEGG enrichment analyses, respectively. The bubble size indicates the number of enriched genes, while the color gradient reflects the significance level of target gene enrichment.

A total of 34 targets for TP were retrieved from the TCMSP database and converted to gene names *via* the UniProt database (S2_Data). Using “Diabetic Nephropathy” as the keyword, 1968 disease-related targets were identified from the GeneCards database, applying a relevance score threshold of ≥5. Additionally, 256 targets were found in the OMIM database and 22 in the TTD database. After merging and removing duplicates, a total of 2173 DKD targets were identified (S3_Data). The intersection of drug and disease targets identified 23 potential targets for TP in treating DKD (Fig. **2A**).

### 4.14 Construction and Analysis of the PPI Network

The 23 potential target genes were entered into the STRING database (species set as “*Homo sapiens*”) for PPI analysis. The results were then imported into Cytoscape for topological analysis and network visualization (Fig. **2B**). Using the Centiscape 2.2 plugin in Cytoscape, seven core targets were identified based on the average values of degree centrality, closeness centrality, and betweenness centrality (Table **4**).

### 4.15 Results of GO and KEGG

A compound-target-disease-pathway interaction network was first constructed using Cytoscape (Fig. **2C**). Subsequently, GO analysis was performed using the DAVID database, yielding 235 GO terms (*P* < 0.05), including 197 BP terms (S4_Data), such as inflammatory response, positive regulation of transcription from RNA polymerase II promoter, and positive regulation of gene expression. Thirteen CC terms (S5_Data) were identified, including nucleoplasm, extracellular region, and cell surface, along with 25 MF terms (S6_Data), such as protein binding, enzyme binding, ubiquitin protein ligase binding, and cytokine activity. The top 10 terms in each category were selected for visualization (Fig. **2D**).

KEGG enrichment analysis identified 101 pathways (*P* < 0.05) (detailed in S7_Data), including pathways in cancer, lipid-atherosclerosis signaling pathway, AGE-RAGE signaling pathway in diabetic complications, IL-17 signaling pathway, and Th17 cell differentiation. The top 20 pathways, ranked by *P*-value, were selected for visualization (Fig. **2E**).

### 4.16 Molecular Docking Validation of Triptolide with Core Targets

Molecular docking of the core targets—IFNG, CXCL8, TNF, TGFB1, IL2, IL4, and RELA—with TP was performed using AutoDock Vina. The binding energy information is provided in Table **4**. According to references [45, 46], a binding energy of < −4.25 kJ/mol indicates moderate binding activity, < −5.00 kJ/mol indicates good binding activity, and < −7.00 kJ/mol indicates strong binding activity. The binding energies of TP with all core target proteins were < −7.00 kJ/mol, suggesting strong binding capabilities. Docking results with binding energies < −8 kJ/mol were visualized. As illustrated in Fig. (**3**), Triptolide engages with the amino acid residues located in the active site pockets of the target proteins *via* hydrogen bonding, van der Waals interactions, and other non-covalent forces.

### 4.17 MR Analysis Results

#### 4.17.1 Instrumental Variables

The study selected seven core genes as exposure factors. The corresponding GWAS data were retrieved from the Open GWAS database (https://gwas.mrcieu.ac.uk/) using the search terms “IFNG (interferon gamma),” “CXCL8 (C-X-C motif chemokine ligand 8),” “TNF (tumor necrosis factor),” “TGFB1 (Transforming growth factor beta-1),” “IL2 (Interleukin-2),” “IL4 (Interleukin-4),” and “RELA (NF-kB subunit).” SNP details are provided in Supplementary Tables **S1** and **S2.1**-**S2.7**. GWAS data for DKD and GFR outcomes were obtained from the GWAS Catalog database (https://www.ebi.ac.uk/gwas/home), and GWAS data for UACR were retrieved from the CKDGen database (http://ckdgen.imbi.uni-freiburg.de/). The selection criteria for instrumental variables were *P* < 5 × 10^−8^ for CXCL8 and *P* < 5 × 10^−6^ for the remaining six core genes. All GWAS data and instrumental variable information are presented in Table **5**, with *F* > 10, indicating strong instrumental variables.

### 4.18 Pleiotropy and Heterogeneity

The MR-Egger intercept values all had *P* > 0.05, indicating no horizontal pleiotropy between the SNPs of the seven core genes and the outcomes DKD, GFR, and UACR. Heterogeneity was assessed using Cochran’s Q test, with all *P*-values exceeding 0.05, suggesting no significant heterogeneity. Therefore, the fixed-effect IVW method (IVW-FE) was employed for analysis. The outcomes of the pleiotropy and heterogeneity analyses are shown in Fig. (**4**).

### 4.19 Two-sample MR Analysis

**Fig. (4):**
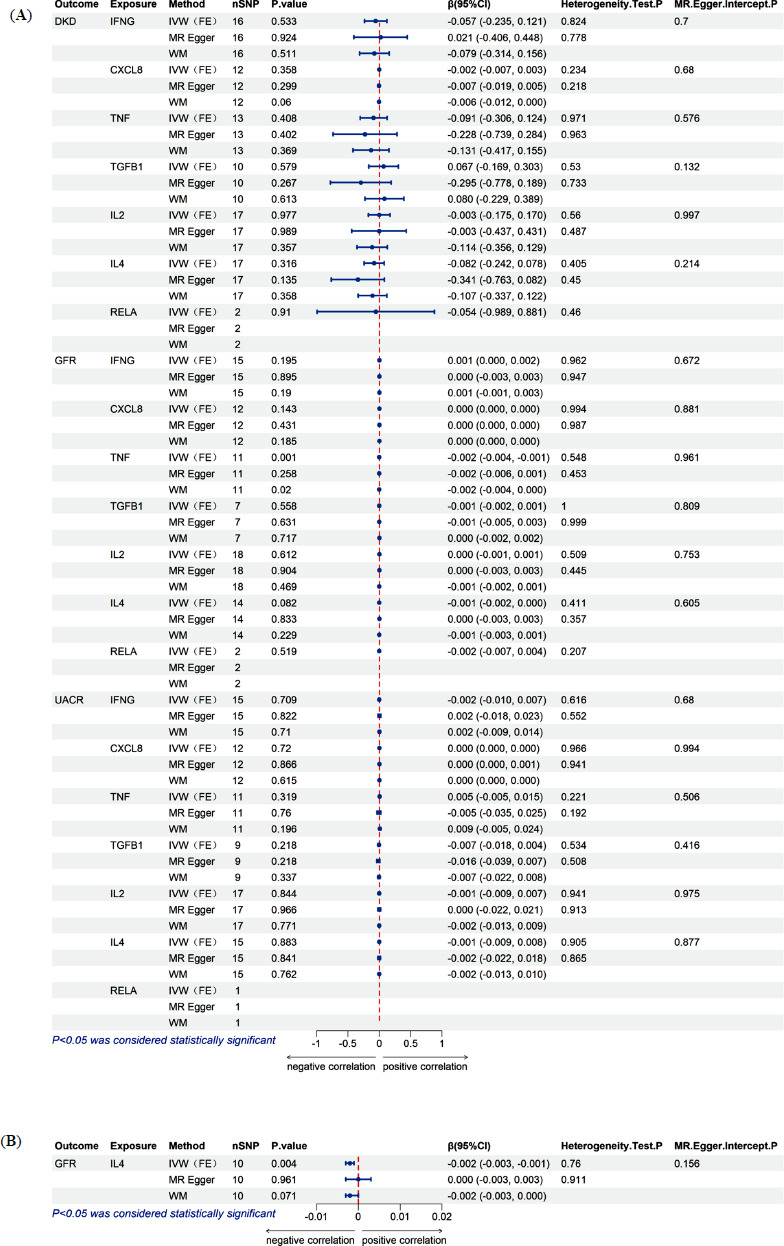
Two-sample MR analysis. (**A**) MR analysis of different exposures and different outcomes; (**B**) MR analysis of IL4 and GFR after removing outlier SNPs.

In this study, two-sample MR analysis was conducted for the seven core genes and the three outcome variables—DKD, GFR, and UACR (detailed in S8_Data). The primary analysis method was the IVW approach. The results showed a significant negative association between TNF and GFR (*β* = -0.002, 95% CI (-0.004, -0.001), *P* = 0.001), while the IVW *P*-values for the other core genes and the three outcomes were all greater than 0.05, suggesting no significant causal relationship between these genes and the outcomes. In the supplementary MR-Egger and WM analyses, the WM method confirmed a consistent negative result regarding the causal relationship between TNF and GFR (*β* = -0.002, 95% CI (-0.004, 0.000), *P* = 0.02). For the other exposure factors and outcomes, the supplementary analyses showed no significant causal relationships (*P* > 0.05) (Fig. **4A**).

Furthermore, based on the sensitivity analysis results, MR analysis, along with pleiotropy and heterogeneity tests, was re-conducted for the exposure factor IL4 and outcome GFR after excluding the outlier SNPs (rs1137193, rs1278107, rs10880857, rs266825). No horizontal pleiotropy or heterogeneity was detected. Using the primary IVW-FE method and supplementary MR-Egger and WM methods, both the IVW-FE and WM methods showed *P*-values less than 0.05 and *β* values less than 0, indicating a significant negative correlation between IL4 and GFR (Fig. **4B**).

### 4.20 Sensitivity Analysis

**Fig. (5):**
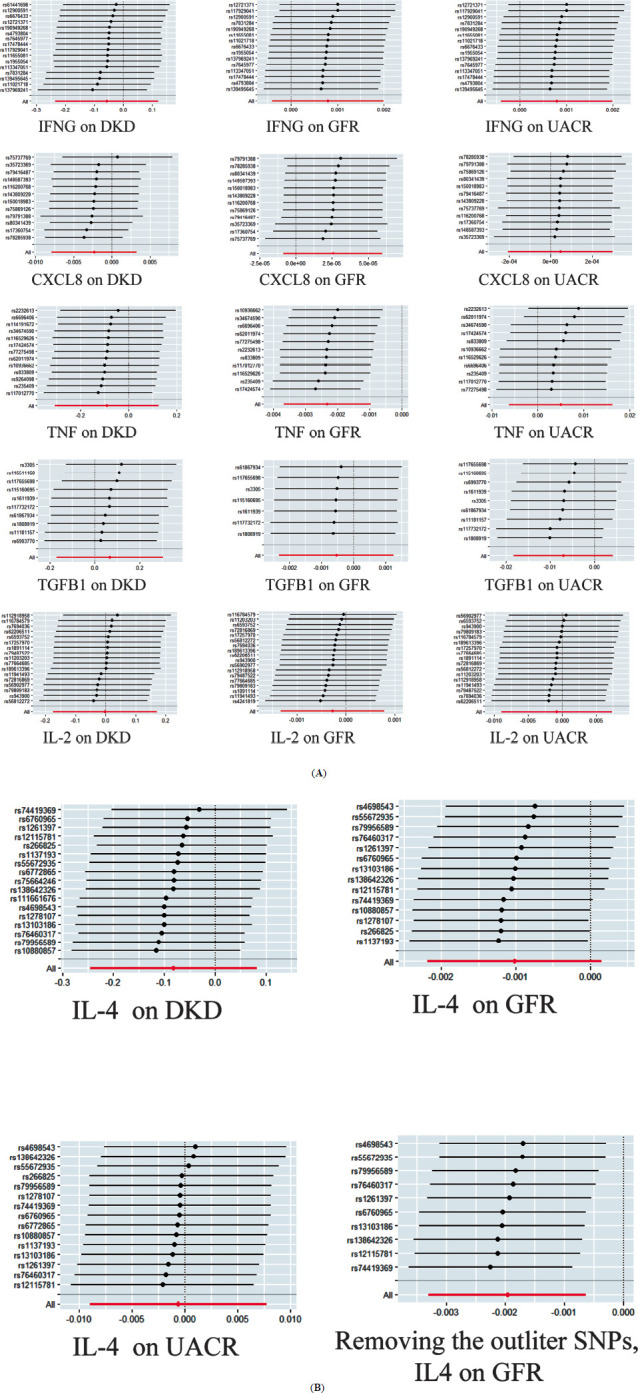
Leave-one-out plots. (**A**) without removing outlier SNPs; (**B**) after removing the outlier SNP, IL4 on GFR.

In the leave-one-out sensitivity analysis of MR results for the seven exposure factors and the three outcomes, only the analysis for IL4 and GFR identified four SNPs that affected effect sizes (rs1137193, rs1278107, rs10880857, rs266825). After excluding these SNPs, no other SNPs significantly impacted the overall effect size. Comparisons among other exposures and outcomes also revealed no SNPs affecting the overall effect, ensuring the robustness of the MR analysis results (Fig. **5**).

## 5 DISCUSSION

### 5.1 Study Significance and efficacy Analysis

Diabetic kidney disease (DKD) is a significant health concern, particularly given its insidious onset, low awareness, high rate of missed diagnoses, and the high risk of cardiovascular complications. It has become one of the leading causes of disability and death in diabetes patients. Once it progresses to the stage of massive proteinuria, the rate at which it advances to ESRD is several times faster than that of other kidney diseases [47]. The current medical treatments for DKD mainly include RAS inhibitors (RASi), SGLT2 inhibitors (SGLT2i), and MRAs. While these medications have demonstrated efficacy in reducing proteinuria, the considerable inter-individual variability in response, coupled with the progressive decline in drug efficacy as renal function deteriorates, underscores the need for more effective therapeutic strategies for DKD [1].

Triptolide (TP), the primary active component of *Tripterygium wilfordii*, has been shown to have significant therapeutic effects in DKD in numerous animal studies [11]. In this study, a meta-analysis was performed to systematically evaluate the role of TP in the treatment of DKD. The results showed that TP significantly reduced kidney weight (KW) and kidney index (KI), suggesting that TP may help delay kidney damage. TP also demonstrated significant efficacy in reducing proteinuria markers such as 24 h UAL, 24 h UTP and UACR, with the most effective dose being 200 < TP ≤ 400 µg/(kg·d). In addition, TP significantly enhanced renal function markers (SCr, BUN) and lipid profiles (TC, TG). The meta-analysis revealed that TP significantly reduced proteinuria, blood lipids, and blood glucose levels, and improved renal function and kidney weight (all *P* < 0.05). Compared with RASi drugs, only one study [41] showed a statistically significant difference in 24 h UTP, suggesting that TP may be more effective than ARB/ACEI drugs in reducing proteinuria. However, further studies are needed to confirm this.

Still, despite its promising therapeutic effects, Triptolide's toxicity, particularly at high doses or with prolonged use, should be considered. Studies show that high doses can lead to liver, kidney, and gastrointestinal toxicity. While low dose triptolide has demonstrated minimal toxicity and a strong anti-fibrotic effect, higher doses may induce oxidative stress and organ damage [48]. Therefore, its clinical application requires balancing efficacy with safety concerns. Advances like nanoparticles (NPs) drug delivery systems show promise in reducing Triptolide's toxicity by enhancing bioavailability and minimizing adverse effects [49, 50], offering an exciting direction for future research.

### 5.2 Molecular Mechanism

Building on the results of the meta-analysis, further network pharmacology was performed. Database searches and PPI network analysis identified IFNG, CXCL8, TNF, TGFB1, IL2, IL4 and RELA as core targets through which TP exerts its effects. The IFNG gene, located on chromosome 12, encodes interferon-gamma (IFN-γ), a pivotal cytokine in host immune defense mechanisms. Studies indicate that elevated IFNG expression enhances IFN-γ secretion, subsequently promoting macrophage polarization and inducing the release of pro-inflammatory cytokines (such as IL-1β and TNF-α), thereby driving the initial inflammatory response in renal fibrosis [51, 52]. Consequently, increased IFNG expression is closely linked to the amplified immune-inflammatory response characteristic of renal fibrosis. In diabetic kidney disease, CXCL8 promotes sustained inflammatory responses by recruiting neutrophils to injury sites, thereby exacerbating glomerular and tubular damage [53]. Furthermore, studies show that CXCL8 inhibitors can significantly reduce glomerulosclerosis, mesangial expansion, and extracellular matrix deposition [54], suggesting that CXCL8 plays a pro-fibrotic role in the progression of DKD and that its inhibition may delay fibrosis progression. The TGFB1 gene encodes transforming growth factor-beta 1 (TGF-β1), the most potent pro-fibrotic factor within the TGF-β family. In renal fibrosis, TGF-β1 expression is significantly upregulated in tubular epithelial and interstitial cells. This elevation in TGF-β1 activates downstream Smad3 signaling, which promotes fibroblast proliferation, excessive extracellular matrix synthesis and deposition, and epithelial-mesenchymal transition, thereby initiating and accelerating the fibrotic process [55, 56]. In addition, IL-2, secreted by Th1 cells, exerts immunomodulatory effects by promoting T cell proliferation and activation, particularly enhancing the generation and function of regulatory T cells (Tregs). Tregs play a protective role in renal fibrosis by suppressing inflammatory responses and preventing tissue damage [57]. The RELA gene encodes nuclear factor-kappa B (NF-κB), another key player in the inflammatory response. In a hyperglycemic environment, the polarization balance of macrophages in renal tissue is disrupted, leading to an increase in pro-inflammatory M1 macrophages that predominates in the early stages of diabetic nephropathy (DN) and activates the NF-κB signaling pathway [58]. Upon activation, NF-κB translocates to the nucleus, upregulating pro-inflammatory cytokine expression and inducing aberrant adhesion molecule secretion, which in turn promotes endothelial cell apoptosis, mesangial matrix deposition, and tubulointerstitial fibrosis, ultimately driving renal fibrosis progression [59-61]. Moreover, TNF has been shown to worsen the progression of DKD by promoting podocyte apoptosis through free cholesterol accumulation, as it reduces ABCA1-mediated cholesterol efflux and SOAT1-mediated cholesterol esterification, resulting in albuminuria [62]. TNF-α also induces the production of several pro-inflammatory cytokines, leading to renal damage [63]. Finally, IL4, produced by Th2 cells, has been implicated in promoting renal fibrosis. Clinical studies have found that IL4 activates STAT6, which promotes the proliferation and differentiation of Th2 cells. These cells secrete more IL4 and IL-13, enhancing the fibrotic process. Additionally, IL4 synergizes with the hyperglycemic environment to upregulate the expression of TGF-β1, fibronectin, and collagen type I, thereby promoting epithelial-mesenchymal transition and extracellular matrix accumulation in tubular epithelial cells, ultimately exacerbating the progression of DKD [64, 65]. However, some studies suggest that IL4 may have anti-inflammatory properties and protective effects on renal function [51, 66].

Building upon Meta-analysis, network pharmacology analysis, and molecular docking validation, this study integrated large-scale GWAS data and employed two-sample MR analysis to further investigate the potential causal relationships between core genes and DKD. Although no individual core gene was found to be directly associated with DKD, a significant negative correlation was observed between the exposure factors TNF and GFR. Additionally, to assess the robustness of our findings, we conducted a “leave-one-out” sensitivity analysis for all exposure-outcome associations. Notably, in the sensitivity analysis between IL4 exposure and GFR outcome, we identified outlier SNPs that significantly affected the effect size. After excluding these outlier SNPs, IL4 still exhibited a significant negative correlation with GFR. A decline in GFR and an increase in UACR are key biomarkers for diagnosing DKD and monitoring the progression of renal impairment [67]. The MR analysis further corroborated the promoting role of TNF and IL4 in the progression of DKD.

Network pharmacology and molecular docking analyses demonstrated that TP exhibits a high binding affinity with both TNF and IL4 proteins, suggesting that TP’s protective effect in DKD may be intricately linked to its modulation of the TNF and IL4 signaling pathways. TNF, particularly TNF-α, is a classical pro-inflammatory cytokine that plays a pivotal role in various immune responses. Previous studies have shown that TNF-α can upregulate the expression of TNFSF10 (encoding the tumor necrosis factor-related apoptosis-inducing ligand, TRAIL) and TNFRSF10B (encoding death receptor 5, DR5). TRAIL, by binding to DR5, activates intracellular death signaling, thereby inducing PANoptosis (a convergence of apoptosis, pyroptosis, and necroptosis) in podocytes, leading to podocyte dysfunction and damage [68]. In contrast, IL4, a critical immune regulatory factor, normally helps modulate immune responses, promote tissue repair, and exert anti-inflammatory effects. However, in the context of chronic immune activation and sustained inflammation, IL4 exacerbates the fibrotic process by enhancing M2 macrophage polarization, promoting the secretion of TGF-β1, and regulating the expression of fibrosis-related genes, ultimately accelerating the fibrotic process [58, 69].

In light of the MR analysis findings, future research should focus on elucidating the specific mechanisms by which TP regulates TNF and IL4 signaling pathways. Notably, systematic *in vivo* and *in vitro* models, coupled with cutting-edge single-cell technologies, should be employed to explore how TP precisely modulates macrophage polarization, inflammatory responses, and the progression of fibrosis. Furthermore, clinical data should be incorporated to further validate TP’s protective role in DKD progression and assess its potential as a targeted therapeutic strategy, providing the necessary theoretical foundation for the development of personalized treatment regimens for DKD.

KEGG pathway enrichment analysis revealed that TP exerts its therapeutic effects in DKD primarily through the lipid-atherosclerosis, AGE-RAGE and IL-17 signaling pathways. Hyperlipidemia leads to lipotoxicity and lipid deposition in the renal tubules, which stimulates mesangial cell proliferation, damages podocytes, increases proteinuria, alters haemodynamics, and results in glomerulosclerosis [70]. In addition, Hyperlipidemia exacerbates insulin resistance, further promoting the development of DKD [71]. Activation of the AGE-RAGE pathway upregulates the Wnt/β-catenin pathway and MAPK pathway, inducing the expression of numerous pro-inflammatory factors, adhesion molecules and growth factors, leading to inflammation, glomerulosclerosis and tubulointerstitial fibrosis [72]. This pathway also exacerbates oxidative stress, damages podocytes and promotes renal fibrosis [72, 73]. The IL-17 pathway, through IL-17 secreted by Th17 cells, increases the expression of pro-inflammatory cytokines and chemokines in renal tubular epithelial and mesangial cells, resulting in macrophage recruitment and exacerbating inflammatory infiltration and kidney damage [74, 75]. Studies have shown that silencing the IL-17 gene reduces albuminuria, and ameliorates glomerular injury, macrophage aggregation and renal fibrosis, thereby preventing the progression of DKD [76].

## 6 LIMITATIONS

In the meta-analysis, firstly, the heterogeneity of the included studies may affect the reliability of the findings. Variations in study design, sample characteristics, and outcome measurements could lead to inconsistent results. Secondly, the total sample size and data quality might influence the robustness of the conclusions. Smaller sample sizes or low-quality data increase the risk of both false-positive and false-negative results. Additionally, publication bias could distort the overall conclusions, especially by introducing systematic biases in effect estimates and causal inferences. Although methods like funnel plots can be used to detect publication bias, it may still impact the validity of the research findings. Finally, the lack of prospective registration of the meta-analysis protocol may increase the risk of selective reporting, whereas registration would improve the transparency of the research process. The predictive results from network pharmacology and molecular docking require experimental validation to confirm their biological significance and clinical feasibility. In MR analysis, genetic background differences could contribute to data heterogeneity, potentially affecting the accuracy of causal inferences. Although we explored the causal relationships between 7 core genes and DKD, environmental factors such as diet and lifestyle may act as confounding variables that influence the results. Despite using the “leave-one-out” sensitivity analysis to enhance the reliability of the results, it remains challenging to completely eliminate all confounding effects.

## CONCLUSION

The meta-analysis results demonstrated that TP has significant efficiency in reducing kidney weight, lowering proteinuria, and improving renal function and lipid profiles. Through network pharmacology and molecular docking analyses, 7 core genes—IFNG, CXCL8, TNF, TGFB1, IL2, IL4, and RELA—were identified, and their mechanisms of action in DKD were revealed. MR analysis further validated the causal relationships between TNF, IL4, and DKD, suggesting that TNF and IL4 may be damaging factors in DKD. KEGG pathway analysis indicated that TP exerts its effects through signaling pathways such as lipid-atherosclerosis, AGE-RAGE, and IL-17. This study highlights the multi-target, multi-pathway, and multi-process nature of TP’s therapeutic action in treating DKD, providing new insights into its potential clinical applications. The identification of key genes such as TNF and IL4 suggests novel therapeutic targets for DKD, which could lead to the development of more effective treatments. Furthermore, the integration of multiple analytical techniques, including meta-analysis, network pharmacology, molecular docking, and MR analysis, enhances the robustness and reliability of the findings, offering a comprehensive view of TP’s mechanisms.

In conclusion, this research contributes to a deeper understanding of TP’s therapeutic potential for DKD, suggesting that TP may serve as a promising candidate for future clinical studies. However, further investigation, including clinical trials, is necessary to confirm these findings and explore the safety and efficacy of TP in human populations. Future research could focus on the development of TP-based formulations that target the identified genes and pathways, which may provide more personalized treatment strategies for DKD patients.

## Figures and Tables

**Fig. (3):**
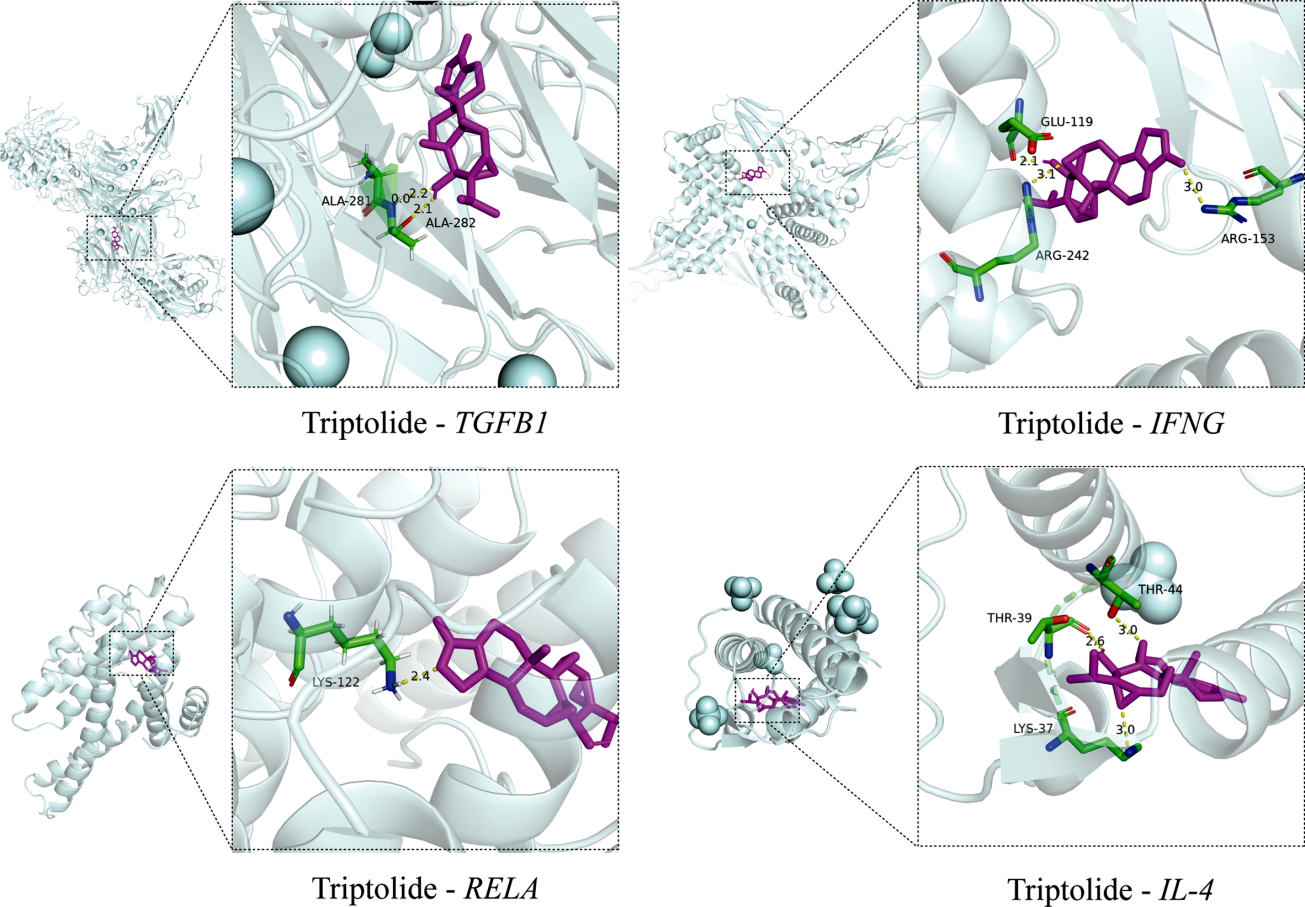
Molecular docking of TP with the core targets of DKD.

**Table 1:** Basic characteristics of the included studies.

**Study Inclusion**	**Animal Characteristics** **(Species, Sex, Age [w], Weight [g])**	**Sample** **Size (T/C)**	**Modeling Method**	**Criteria for successful model establishment**	**Intervention** **Measures**		**Intervention Dose** **(mg/Kg/d)**	**Intervention Duration (w)**	**Outcomes**
					**T**	**C**			
Ren *et al*. 2020 [20]	BKS-db/ db mice, male, 9, 38.25 ± 1.42	6/6	NM	NM	TP	NS	0.05	8	c, f, h, i, j
	ibid.	6/6	NM	NM	TP	Telmisartan	T: 0.05; C:5	8	c, f, h, i, j
Zhu *et al*. 2014 [21]	SD rats, male, 8, 180 ± 20	44/22	STZi (60 mg/Kg)	72 h post-STZi; FBG ≥ 16.7 mmol/L; Urine glucose “+++” >3 days.	TP	NS	0.2/0.4	4/8/12	b, c, d, f, h, i, j
Xue *et al*. 2018 [22]	SD rats, male, 6, 160 ± 8	15/15	HFD 8 w+ STZi (30 mg/Kg)	1 week post-STZi; Random glucose ≥ 16.7 mmol/L twice	TP	NS	0.2	12	a, c, d, f, h, i, j
Han 2018 [23]	SD rats, male, 6, 170 ± 10	10/10	HFD 8 w+STZi (30 mg/Kg)	1) 3d, 5d, and 7d post-STZi; Random glucose ≥ 16.7 mmol/L twice; 2) Week 6 post-DM: 24 h UMA ↑ *vs.* control.	TP	DMSO	0.1	12	a, c, d, f, j
Fan *et al*. 2018 [24]	C57BL/6-Ins2Akitamice, male, 24, NM	24/8	NM	NM	TP	NS	0.025/0.05/0.1	8	b, c, d, g, h, i, j
Wang *et al*. 2017 (1) [25]	SD rats, male, NM, 190 ± 20	10/10	STZi (65 mg/Kg)	1 week post-STZi; FBG > 16.7 mmol/L; Urine glucose “+++” > 3 days	TP	NS	0.2	8	a, c, d, e, j
Wang *et al*. 2017(2) [26]	SD rats, male, NM, 190 ± 20	10/10	STZi (65 mg/Kg)	1 week post-STZi; FBG > 16.7 mmol/L; Urine glucose “+++” > 3 days	TP	NS	0.2	8	a, c, d, e, j
Wang *et al*. 2017(3) [27]	SD rats, male, NM, 190 ± 20	10/10	STZi (65 mg/Kg)	1 week post-STZi; FBG > 16.7 mmol/L; Urine glucose “+++” > 3 days	TP	NS	0.2	8	a, c, d, e, j
An *et al*. 2017 [28]	SD rats, male, 8, 200-250	20/9	HFD 4 w+ STZi (60 mg/Kg)	1) 72 h post-STZi; Blood glucose ≥ 16.7 mmol/L; Significant polyuria; 2) 4 weeks post-modeling, 24 h UMA excretion increased to (400.56 ± 97.93) µg/24 h, confirming DN model success	TP	NS	0.2/0.4	4	f, j
-	ibid.	20/10	ibid.	ibid.	TP	Benazepril	T:0.2/0.4 C:10	4	f, j
You *et al*. 2015 [29]	SD rats, male, NM, 160-180	13/13	STZi (52 mg/Kg/d) for 5 days	72 h post-STZi; FBG ≥ 16.7 mmol/L; Urine glucose “+++”	TP	water	NM	4/8	f
Ye *et al*. 2015 [30]	SD rats, male, NM, NM	44/22	STZi (60 mg/Kg)	72 h post-STZi; FBG ≥ 16.7 mmol/L; Urine glucose “+++” > 3 days	TP	NS	0.2/0.4	4/8/12	b, f, j
Li *et al*. 2015 [31]	SD rats, male, NM, 230 ± 20	15/15	HFD 4 w+ STZi (30 mg/Kg)	5 days post-STZi; FBG ≥ 16.7 mmol/L	TP	Vegetableoil	0.2	4	f, j
Ruan *et al*. 2014 [32]	SD rats, male, NM, 200 ± 20	18/18	HFD 8 w+ STZi (30 mg/Kg)	NM	TP	Sterile water	0.2	8/12	a, c, d, g, h, i, j
Liu *et al*. 2014 [33]	SD rats, male, NM, 180~200	16/16	STZi (60 mg/Kg)	72 h post-STZi; FBG ≥ 16.7 mmol/L	TP	NS	0.2	4/8	c, d, f,
Li 2014 [34]	SD rats, male, 8, 200 ± 20	16/16	HFD 8 w+ STZi (30 mg/Kg)	1 week post-STZi; Non-fasting glucose ≥ 16.7 mmol/L; Insulin resistance	TP	NS	0.2	8	a, c, d, g, h, i, j
Li *et al*. 2013 [35]	SD rats, male, 8, 200 ± 20	8/8	HFD 8 w+ STZi (30 mg/Kg)	1 week post-STZi; Non-fasting glucose ≥ 16.7 mmol/L; Insulin resistance	TP	NS	0.2	8	a, c, d, g, h, i, j
	ibid.	8/8	ibid.	ibid.	TP	Telmisartan	T:0.2 C:50	8	a, c, d, g, h, i, j
Ma *et al*. 2012 [36]	Wistar rats, male, NM, 200 ± 20	12/11	HFD 8 w+ STZi (30 mg/Kg)	NM	TP	DMSO	0.2	8	a, c, d, g, h, i
	ibid.	12/12	ibid.	NM	TP	Irbesartan	T:0.2 C:50	8	a, c, d, g, h, i
Ma *et al*. 2010 [37]	Wistar rats, male, 8, 200 ± 20	14/14	HFD 8 w+ STZi (30 mg/Kg)	1 week post-STZi; FBG ≥ 10.0 mmol/L; Insulin sensitivity ≤ normal	TP	Equal solvent	0.2	8	a, c, g, h, i, j
Ma *et al*. 2009 [38]	Wistar rats, male, NM, 200 ± 20	12/12	HFD 8 w+ STZi (30 mg/Kg)	1 week post-STZi; Non-fasting glucose ≥ 16.7 mmol/L; Insulin resistance	TP	NS	0.2	8	a, b, g, h, i, j
Ma *et al*. 2008 [39]	Wistar rats, male, NM, 200 ± 20	7/7	HFD 4 w+ STZi (30 mg/Kg)	1 week post-STZi; Non-fasting glucose ≥ 16.7 mmol/L; Higher blood pressure and lipids; Insulin resistance.	TP	NS	0.2	12	b, g, h, i, j
Gao *et al*. 2009 [40]	db/db mice, equally divided by sex, 9, NM	36/18	NM	NM	TP	NS	0.025/ 0.05	4/8/12	f, h, i, j
	ibid.	36/18	ibid.	ibid.	TP	Valsartan	T:0.025/0.05 C:20	4/8/12	f, h, i, j
Dong *et al.* 2017 [41]	SD rats, male, 8, 200-250	24/12	HFD 4 w+ STZi (60 mg/Kg)	3 days post-STZi; FBG ≥ 16.7 mmol/L; Polyuria symptoms	TP	NS	0.2/0.4	4	e, j
	ibid.	24/12	ibid.	ibid.	TP	Benazepril	T:0.2/0.4 C:10	4	e, j
Liu *et al*. 2024 [42]	C57 BLKS-db/db mice, male, 7, NM	6/6	NM	NM	TP	DMSO	0.05	12	c, d, g
Guo *et al*. 2016 [43]	SD rats, male, 5-6, NM	45/15	HFD 4 w+ STZi (30 mg/Kg)	Blood glucose > 16.7 mmol/L	TP	distilled water	6/12/24	4	a, c, d, f, h, i, j
Ren *et al*. 2022 [14]	C57BL/ Ksjsdb/db mice, male, 8, NM	12/6	NM	NM	TP	NS	0.05/0.075	12	c, d, f, h, i, j
	ibid.	12/6	ibid.	ibid.	TP	Telmisartan	T:0.05/0.075 C:5	12	c, d, f, h, i, j
Lv *et al*. 2023 [11]	C57BL/6J mice, male, 6-8, NM	6/6	HFD 2 months+ STZi (50 mg/Kg) for 7 days	7 days post-STZi; FBG ≥ 16.7 mmol/L; 24 h UMA ↑	TP	DMSO	0.1	12	a, c, f, j
Pang *et al*. 2021 [44]	SD rats, male, NM, 180-220	9/3	HFD 4 w+ STZi (35 mg/Kg)	72 h post-STZi; FBG > 13.9 mmol/L; Random glucose > 16.7 mmol/L	TP	NS	0.2/0.3/0.4	4	a, d, h, i

**Abbreviations**: NM: No Mention; T: Test group; C: Control group; STZi: Streptozotocin injection; TP: Triptolide; NS: Normal Saline; HFD: High fat diet; FBG: Fasting blood-glucose; DM: Diabetes Mellitus; DKD: Diabetic Kidney Disease; UAM: Urinary Microalbumin. a: Kidney index, b: kidney weight, c: Serum creatinine, d: Blood urea nitrogen, e: 24-hour urinary protein quantity, f: 24-hour urine albumin, g: urinary albumin-to-creatinine ratio, h: total cholesterol, i: triglyceride, j: fasting blood glucose or random blood glucose.

**Table 2:** Comparison of clinical outcomes between groups.

**Outcome Measures**		**Cases (T/C)**	**Heterogeneity Test**		**SMD**	**95%CI**	***P*-value**	**References**
			***I^2^*/%**	***P*-value**				
Renal weight	KW	103/103	78	<0.00001	-0.71	[-1.41, -0.02]	0.04	[21, 24, 30, 38, 39]
	KI	186/185	86	<0.00001	-3.61	[-4.50, -2.72]	<0.00001	[11, 22, 23, 25-27, 32, 34-38, 43]
Proteinuria	24 h UAL	267/265	82	<0.00001	-3.69	[-4.41, -2.98]	<0.00001	[11, 14, 20-22, 28-31, 33, 40, 43]
	24 h UTP	50/48	0	1.00	-3.12	[-3.75, -2.49]	<0.00001	[25-27, 41]
	UACR	117/116	94	<0.00001	-4.73	[-6.70, -2.76]	<0.00001	[24, 32, 34-39, 42]
Renal function	SCr	277/276	79	<0.00001	-0.89	[-1.34, -0.44]	<0.0001	[11, 14, 20-27, 32-37, 42-44]
	BUN	221/220	78	<0.00001	-0.96	[-1.43, -0.48]	<0.0001	[14, 21-24, 32-36, 42-44]
Lipid	TC	264/263	71	<0.00001	-1.33	[-1.73, -0.93]	<0.00001	[14, 20-22, 24, 32, 34-40, 43, 44]
	TG	264/263	77	<0.00001	-1.47	[-1.92, -1.01]	<0.00001	[14, 20-22, 24, 32, 34-40, 43, 44]
BG	FBG/RBG	374/374	75	<0.00001	-0.55	[-0.87, -0.22]	0.001	[11, 14, 20-28, 30-32, 34, 35, 37-41, 43]

**Abbreviations**: BG: blood glucose; FBG: fasting blood glucose; RBG: random blood glucose.

**Table 3:** Comparison of clinical outcomes between TP and ARB/ACEI drugs.

**Outcome Measures**		**Cases (T/C)**	**Heterogeneity Test**		**SMD**	**95%CI**	***P*-value**	**References**
			***I^2^*/%**	***P*-value**				
Proteinuria	24 h UAL	74/74	40	0.09	-0.24	[-0.68, 0.20]	0.28	[14, 20, 28, 40]
	24 h UTP	20/20	0	0.50	-1.17	[-1.85, -0.48]	0.00008	[41]
	UACR	20/20	0	0.65	-0.24	[-0.50, 0.01]	0.06	[35, 36]
Renal function	SCr	38/38	0	0.55	-0.21	[-0.67, 0.25]	0.37	[14, 20, 35, 36]
	BUN	32/32	0	0.65	-0.50	[-1.01, 0.00]	0.05	[14, 35, 36]

**Note**: “-” means that *I^2^* test is not required.

**Table 4:** Core targets and their topological analysis and molecular docking.

**Core Target**	**Topological Analysis**			**Molecular Docking**		
	**Betweenness**	**Closeness**	**Degree**	**Ligand**	**Receptor Protein**	**Binding Energy (kJ/mol)**
IFN-γ	11.686	0.048	42	triptolide	1FYH	-8.3
CXCL8	11.686	0.048	42		1QE6	-7.7
TNF	11.686	0.048	42		7KPA	-7.7
TGFB1	8.043	0.045	40		6OM2	-8.9
IL2	4.586	0.045	40		7M2G	-7.5
IL4	3.660	0.043	38		2B8Y	-8.1
RELA	3.660	0.043	38		6QHL	-8.9

**Table 5:** Description of GWAS data and instrumental variables in this study.

**Year**	**Exposure/Outcome**	**Population**	**Sample Size**	**Database**	**GWAS ID/PMID**	**IVs**	**F-value**
2018	IFNG	European	3,301	Open GWAS	prot-a-1429	17	23.38
2018	CXCL8	European	3,394	Open GWAS	prot-b-11	12	132.96
2018	TNF	European	3,301	Open GWAS	prot-a-3029	13	23.35
2018	TGFB1	European	3,301	Open GWAS	prot-a-2962	10	22.83
2018	IL2	European	3,301	open GWAS	prot-a-1512	19	25.47
2018	IL4	European	3,301	Open GWAS	prot-a-1532	17	23.94
2018	RELA	European	31,684	Open GWAS	eqtl-a-ENSG00000173039	2	31.11
2021	DKD	452280 European, 132984 East Asian	585264	GWAS Catolog	GCST90018832	-	-
2021	GFR	European	1,004,040	GWAS Catolog	GCST90103634	-	-
2019	UACR	European	192868	CKD Gen	PMID: 31511532	-	-

**Abbreviation**: IVs: Instrumental variables.

## Data Availability

All data generated or analyzed during this study are included in this published article.

## LIST OF ABBREVIATIONS

ACEIs: Angiotensin-converting Enzyme Inhibitors
BP: Biological Processes
CC: Cellular Components
DKD: Diabetic Kidney Disease
ESRD: End-stage Renal Disease
eGFR: estimated Glomerular Filtration Rate
GO: Gene Ontology
GWAS: Genome-wide Association Studies
GFR: Glomerular Filtration Rate
IVs: Instrumental Variables
MR: Mendelian Randomization
PDB: Protein Data Bank
PPI: Protein-protein Interaction
RASi: Renin-angiotensin System Inhibitors
SNPs: Single Nucleotide Polymorphisms
TP: Triptolide
UACR: Urinary Albumin-to-creatinine Ratio
