# Supplementary material for: Comprehensive Evaluation of Triptolide’s Therapeutic Mechanisms in Diabetic Kidney Disease via Meta-Analysis, Network Pharmacology, Molecular Docking, and Mendelian Randomization
Source: Curr Pharm Des. 2025 Jul 23;32(10):791–810. doi: 10.2174/0113816128367671250714100708 (PMC13223498; doi:10.2174/0113816128367671250714100708)
Supplement: Supplementary file 1 [file CPD-32-10-791_SD1.pdf]

Supplementary Material

Comprehensive Evaluation of Triptolide's Therapeutic Mechanisms in Diabetic Kidney Disease *via* Meta-Analysis, Network Pharmacology, Molecular Docking, and Mendelian Randomization

Jing Ni[1,2], Siyuan Song[2,4], Yi Wei[2,4], Qiling Zhang[2,4], Wei Li[1,3] and Jiangyi Yu[2,4,*]

[1]*Department of Endocrinology, Jiangning District of Nanjing Chinese Medicine Hospital, Affiliated Jiangning Hospital of Chinese Medicine, China Pharmaceutical University, Nanjing, China;* [2]*Nanjing University of Chinese Medicine, Nanjing, China;* [3]*Department of Neurology, Jiangning District of Nanjing Chinese Medicine Hospital, Affiliated Jiangning Hospital of Chinese Medicine, China Pharmaceutical University, Nanjing, China;* [4]*Department of Endocrinology, Jiangsu Provincial Hospital of Chinese Medicine, Affiliated Hospital of Nanjing University of Chinese Medicine, Nanjing, China*

1873-4286/26 **© 2026 Bentham Science Publishers**

	DKD+TP			DKD				Std. Mean Difference
Study or Subgroup	Mean	SD	Total	Mean	SD	Total	Weight	IV, Random, 95% CI
**2.4.1 STZ**								
Liu Q, et al 2014 A	11.05	7.71	8	13.18	2.44	8	6.5%	-0.35 [-1.34, 0.64]
Liu Q, et al 2014 B	8.13	7.28	8	15.35	1.8	8	6.4%	-1.29 [-2.39, -0.18]
Ye X, et al 2015 D	6.6	0.5	5	11.6	0.7	5	2.8%	-7.42 [-11.78, -3.06]
Ye X, et al 2015 A	8.9	1.8	5	11.6	0.7	5	5.9%	-1.79 [-3.38, -0.19]
Ye X, et al 2015 B	7.5	0.4	5	12.1	0.6	5	2.5%	-8.15 [-12.90, -3.40]
Ye X, et al 2015 C	6.6	0.7	5	13.4	1.5	5	3.9%	-5.25 [-8.45, -2.04]
Ye X, et al 2015 E	6	0.1	5	12.1	0.6	5	1.4%	-12.81 [-20.13, -5.49]
Ye X, et al 2015 F	5.1	0.3	5	13.4	1.5	5	3.0%	-6.93 [-11.02, -2.84]
You LJ, et al 2015 A	4.64	0.87	13	5.86	0.56	13	6.6%	-1.61 [-2.52, -0.71]
You LJ, et al 2015 B	2.26	0.68	13	8.23	0.16	13	3.5%	-11.70 [-15.24, -8.17]
Zhu JJ, et al 2014 A	4.64	0.87	13	5.86	0.56	13	6.6%	-1.61 [-2.52, -0.71]
Zhu JJ, et al 2014 B	2.26	0.68	13	8.23	0.16	13	3.5%	-11.70 [-15.24, -8.17]
Zhu JJ, et al 2014 C	7.94	0.45	5	10.7	0.75	5	4.6%	-4.03 [-6.62, -1.45]
Zhu JJ, et al 2014 D	7.12	0.29	5	12.1	0.41	5	1.4%	-12.67 [-19.91, -5.43]
Zhu JJ, et al 2014 E	6.5	0.5	5	13.42	1.61	5	3.9%	-5.24 [-8.44, -2.04]
Zhu JJ, et al 2014 F	6.58	0.59	5	10.7	0.75	5	3.7%	-5.51 [-8.86, -2.17]
**Subtotal (95% CI)**			**118**			**118**	**66.3%**	**-5.00 [-6.44, -3.55]**
Heterogeneity: Tau² = 6.01; Chi² = 120.26, df = 15 (P < 0.00001); I² = 88%								
Test for overall effect: Z = 6.79 (P < 0.00001)								
**2.4.2 STZ+HFD**								
Guo H， et al 2016 A	381.75	294.52	15	1,311.03	795.21	15	6.7%	-1.51 [-2.33, -0.68]
Guo H， et al 2016 B	214.45	229.73	15	1,311.03	795.21	15	6.6%	-1.82 [-2.69, -0.95]
Guo H， et al 2016 C	547.53	376.99	15	1,311.03	795.21	15	6.7%	-1.19 [-1.98, -0.41]
Han F 2018	148.23	14.22	10	237.56	15.81	10	5.1%	-5.69 [-7.84, -3.54]
Lv CL. et al 2023	88.47	9.59	6	171.4	0.83	6	2.0%	-11.25 [-16.85, -5.64]
Xue M, et al 2018	159	29.3	15	243.6	35.1	15	6.5%	-2.55 [-3.54, -1.55]
**Subtotal (95% CI)**			**76**			**76**	**33.7%**	**-2.59 [-3.72, -1.46]**
Heterogeneity: Tau² = 1.39; Chi² = 28.19, df = 5 (P < 0.0001); I² = 82%								
Test for overall effect: Z = 4.49 (P < 0.00001)								
**Total (95% CI)**			**194**			**194**	**100.0%**	**-4.02 [-4.97, -3.07]**
Heterogeneity: Tau² = 3.33; Chi² = 149.12, df = 21 (P < 0.00001); I² = 86%								
Test for overall effect: Z = 8.31 (P < 0.00001)								
Test for subgroup differences: Chi² = 6.62, df = 1 (P = 0.01), I² = 84.9%								

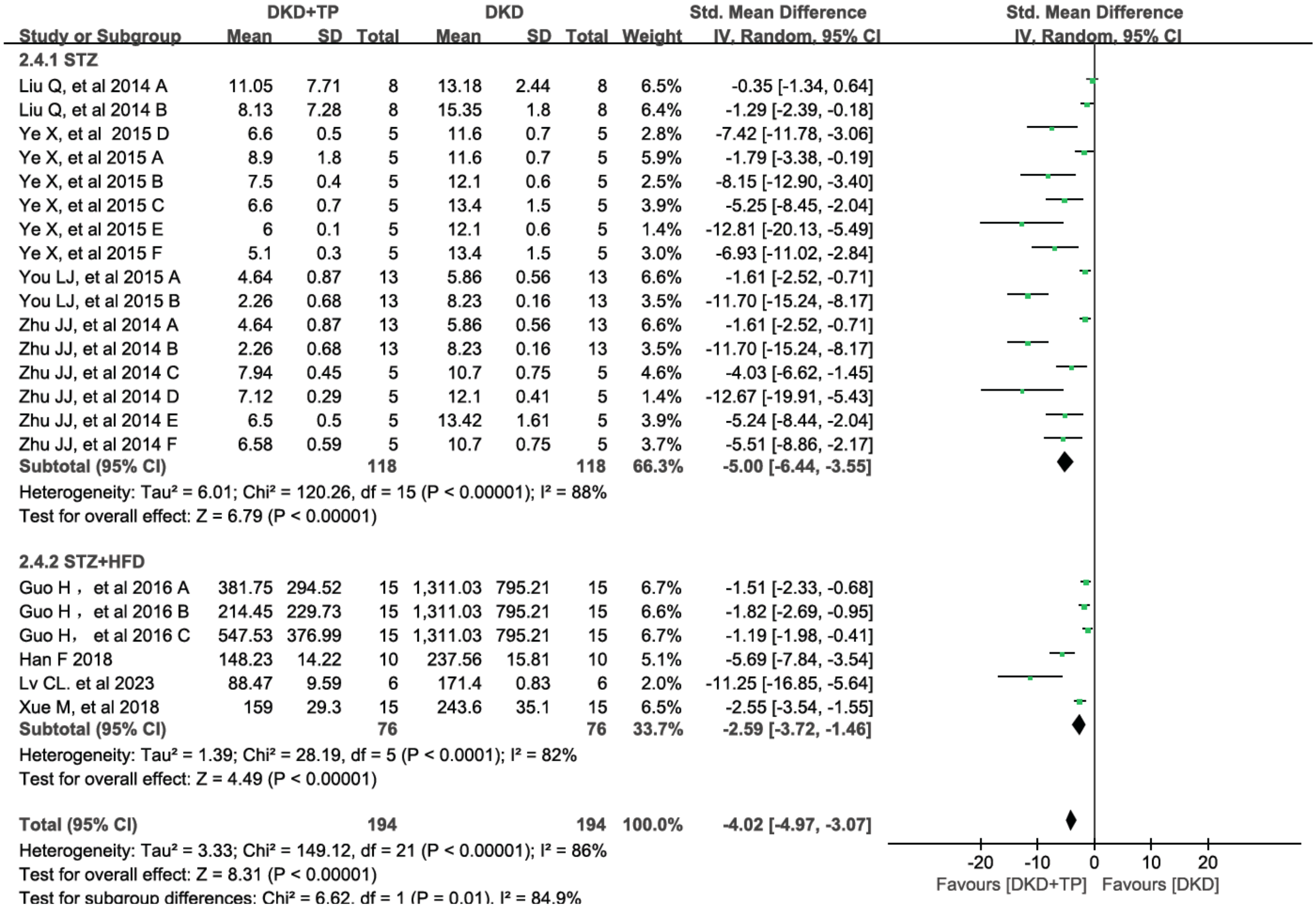

Fig. (S1). Fig. Subgroup analysis of TP’s effect on 24hUAL in DKD with different diabetes induction methods.

Study or Subgroup	DKD+TP Mean	DKD+TP SD	DKD+TP Total	DKD Mean	DKD SD	DKD Total	Weight	Std. Mean Difference IV, Random, 95% CI
**2.2.1 TP≤100ug/(Kg·d)**								
Fan HR, et al 2018 A	6	1.31	8	6.13	3.09	8	3.6%	-0.05 [-1.03, 0.93]
Fan HR, et al 2018 B	6.88	2.17	8	6.13	3.09	8	3.6%	0.27 [-0.72, 1.25]
Fan HR, et al 2018 C	6.75	1.91	8	6.13	3.09	8	3.6%	0.23 [-0.76, 1.21]
Han F 2018	31.98	1.91	10	32.52	1.16	10	3.7%	-0.33 [-1.21, 0.56]
Liu F, et al 2024	13.23	0.45	6	16.12	0.12	6	0.9%	-8.10 [-12.21, -3.99]
Lv CL. et al 2023	78.37	5.84	6	132.86	9.54	6	1.3%	-6.36 [-9.66, -3.06]
Ren LY et al 2022 A	34	7.14	6	45.47	18.6	6	3.3%	-0.75 [-1.94, 0.44]
Ren LY et al 2022 B	31.28	6.48	6	45.47	18.6	6	3.3%	-0.94 [-2.16, 0.28]
Ren LY, et al 2020 A	38.82	3.66	6	45.32	7.63	6	3.3%	-1.00 [-2.24, 0.23]
**Subtotal (95% CI)**			**64**			**64**	**26.5%**	**-0.89 [-1.73, -0.05]**
Heterogeneity: Tau² = 1.10; Chi² = 31.77, df = 8 (P = 0.0001); I² = 75%								
Test for overall effect: Z = 2.07 (P = 0.04)								
**2.2.2 100<TP≤200ug/(Kg·d)**								
LI KZ 2014	71.25	1.3	16	73.44	1.11	16	3.8%	-1.77 [-2.60, -0.93]
Li KZ, et al 2013 A	65.25	7.63	8	73.35	8.62	8	3.5%	-0.94 [-1.99, 0.11]
Liu Q, et al 2014 A	73.27	8.28	8	69.59	9.04	8	3.6%	0.40 [-0.59, 1.39]
Liu Q, et al 2014 B	61.32	7.69	8	76.1	3.92	8	3.1%	-2.29 [-3.63, -0.95]
Ma RX, et al 2012 A	78.62	2.96	12	80.46	3.62	11	3.8%	-0.54 [-1.37, 0.30]
Ma RX, Liu XM, et al 2010	78.6	2	14	80.4	3.6	14	3.8%	-0.60 [-1.36, 0.16]
Pang, RY, et al 2021 A	59.72	3.15	3	72.2	2.49	3	1.1%	-3.52 [-7.27, 0.24]
Ruan J, et al 2014 A	68.8	1.8	9	77.2	3.9	9	3.1%	-2.63 [-3.98, -1.29]
Ruan J, et al 2014 B	67.25	4.1	9	79.3	1.2	9	2.7%	-3.80 [-5.48, -2.12]
Wang DN, Yu R, 2017（2）	110.58	10.22	10	148.35	12.06	10	3.0%	-3.24 [-4.66, -1.81]
Wang DN, Yu R, 2017（1）	110.56	10.21	10	149.38	11.88	10	3.0%	-3.36 [-4.81, -1.90]
Wang DN, Yu R, 2017（3）	112.56	10.01	10	148.25	11.18	10	3.0%	-3.22 [-4.64, -1.80]
Xue M, et al 2018	38.1	4.9	15	37.8	4.6	15	3.9%	0.06 [-0.65, 0.78]
Zhu JJ, et al 2014 A	41.4	8.5	5	32.1	7.1	5	3.1%	1.07 [-0.31, 2.45]
Zhu JJ, et al 2014 B	42.9	7.8	5	37.3	4.5	5	3.2%	0.79 [-0.52, 2.11]
Zhu JJ, et al 2014 C	38.9	2.1	5	36.6	4.9	5	3.2%	0.55 [-0.73, 1.83]
**Subtotal (95% CI)**			**147**			**146**	**50.8%**	**-1.29 [-2.01, -0.57]**
Heterogeneity: Tau² = 1.70; Chi² = 93.54, df = 15 (P < 0.00001); I² = 84%								
Test for overall effect: Z = 3.51 (P = 0.0005)								
**2.2.3 200<TP≤400ug/(Kg·d)**								
Pang, RY, et al 2021 B	54.65	4.59	3	72.2	2.49	3	1.0%	-3.80 [-7.81, 0.20]
Pang, RY, et al 2021 C	52.75	2.63	3	72.2	2.49	3	0.5%	-6.08 [-12.16, 0.01]
Zhu JJ, et al 2014 D	39.2	5.4	5	32.1	7.1	5	3.1%	1.02 [-0.35, 2.38]
Zhu JJ, et al 2014 E	40.2	4.3	5	37.3	4.5	5	3.2%	0.60 [-0.69, 1.88]
Zhu JJ, et al 2014 F	39.1	2.9	5	36.6	4.9	5	3.2%	0.56 [-0.72, 1.84]
**Subtotal (95% CI)**			**21**			**21**	**11.0%**	**0.05 [-1.27, 1.37]**
Heterogeneity: Tau² = 1.11; Chi² = 9.49, df = 4 (P = 0.05); I² = 58%								
Test for overall effect: Z = 0.07 (P = 0.95)								
**2.2.4 TP>400ug/(Kg·d)**								
Guo H， et al 2016 A	37	9.5	15	40	8.8	15	3.9%	-0.32 [-1.04, 0.40]
Guo H， et al 2016 B	40.6	5.1	15	40	8.8	15	3.9%	0.08 [-0.63, 0.80]
Guo H， et al 2016 C	39.6	6.3	15	40	8.8	15	3.9%	-0.05 [-0.77, 0.66]
**Subtotal (95% CI)**			**45**			**45**	**11.7%**	**-0.10 [-0.51, 0.32]**
Heterogeneity: Tau² = 0.00; Chi² = 0.62, df = 2 (P = 0.73); I² = 0%								
Test for overall effect: Z = 0.45 (P = 0.65)								
**Total (95% CI)**			**277**			**276**	**100.0%**	**-0.89 [-1.34, -0.44]**
Heterogeneity: Tau² = 1.23; Chi² = 155.46, df = 32 (P < 0.00001); I² = 79%								
Test for overall effect: Z = 3.88 (P = 0.0001)								
Test for subgroup differences: Chi² = 9.59, df = 3 (P = 0.02), I² = 68.7%								

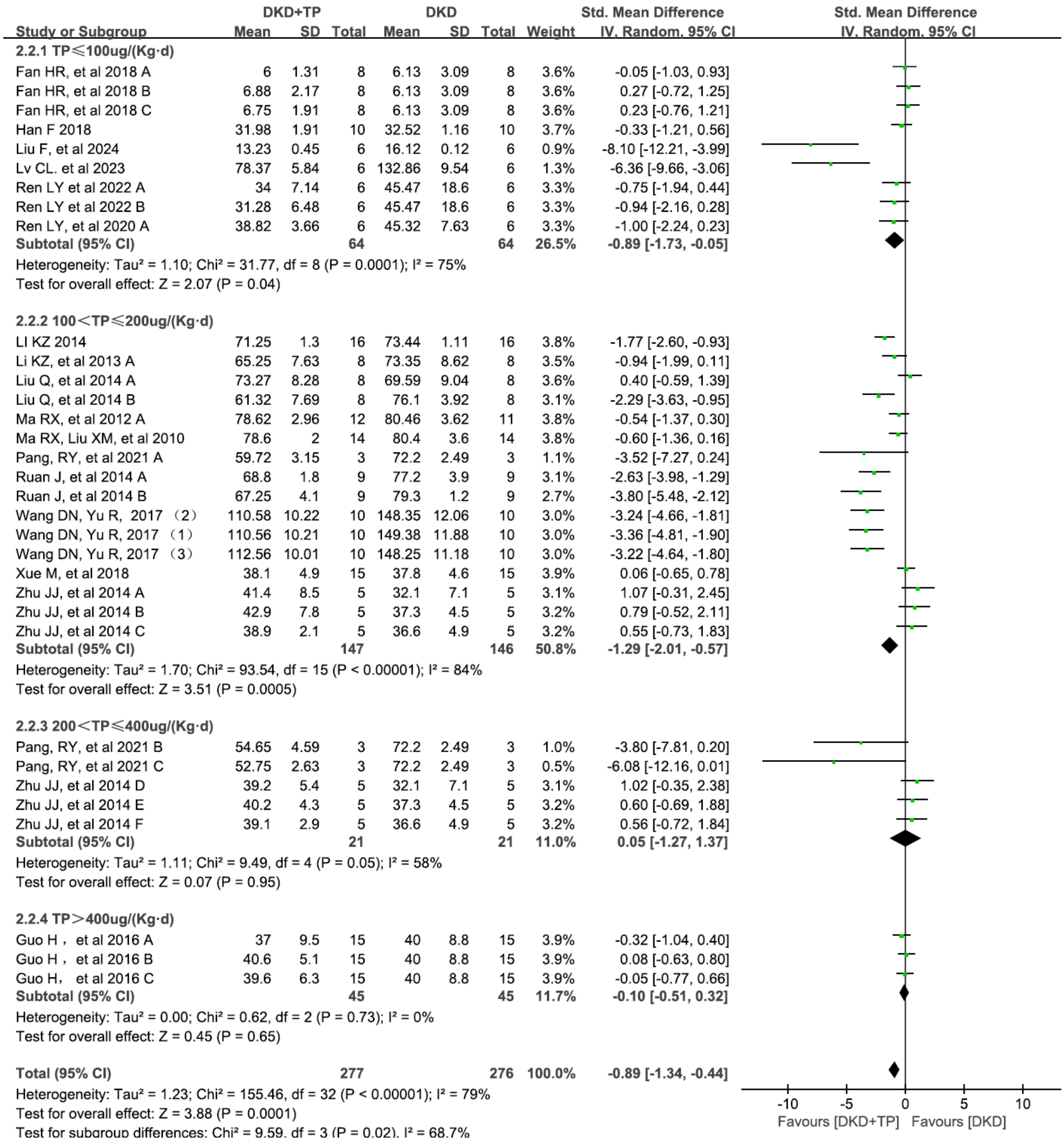

Fig. (S2). Fig. Subgroup analysis of the effect of different doses of TP on SCr in DKD.

	DKD+TP			DKD				Std. Mean Difference
**Study or Subgroup**	**Mean**	**SD**	**Total**	**Mean**	**SD**	**Total**	**Weight**	**IV, Random, 95% CI**
**2.3.1 TP≤100ug/(Kg·d)**								
Fan HR, et al 2018 A	13.47	1.86	8	15.1	2.73	8	4.1%	-0.66 [-1.67, 0.35]
Fan HR, et al 2018 B	15.15	3.02	8	15.1	2.73	8	4.2%	0.02 [-0.96, 1.00]
Fan HR, et al 2018 C	15.74	2.86	8	15.1	2.73	8	4.2%	0.22 [-0.77, 1.20]
Han F 2018	6.94	0.49	10	7.14	0.95	10	4.3%	-0.25 [-1.13, 0.63]
Liu F, et al 2024	8.46	0.27	6	10.47	0.15	6	0.9%	-8.50 [-12.79, -4.20]
Ma RX, et al 2012 A	8.28	1.37	12	9.25	2.02	11	4.4%	-0.55 [-1.38, 0.29]
Ren LY et al 2022 A	5.9	2.41	6	9.86	8.53	6	3.9%	-0.58 [-1.75, 0.58]
Ren LY et al 2022 B	4.18	2.52	6	9.86	8.53	6	3.8%	-0.83 [-2.04, 0.37]
**Subtotal (95% CI)**			**64**			**63**	**29.8%**	**-0.51 [-1.12, 0.09]**
Heterogeneity: Tau² = 0.42; Chi² = 16.89, df = 7 (P = 0.02); I² = 59%								
Test for overall effect: Z = 1.66 (P = 0.10)								
**2.3.2 100＜TP≤200ug/(Kg·d)**								
LI KZ 2014	8.51	0.22	16	9.01	0.27	16	4.4%	-1.98 [-2.84, -1.11]
Li KZ, et al 2013 A	8.99	1.32	8	11.23	1.09	8	3.8%	-1.75 [-2.95, -0.55]
Liu Q, et al 2014 A	31.33	9.55	8	24.11	3.59	8	4.1%	0.95 [-0.10, 2.00]
Liu Q, et al 2014 B	25.03	6.5	8	34.86	2.17	8	3.7%	-1.92 [-3.16, -0.67]
Ma RX, et al 2012 A	8.28	1.37	12	9.25	2.02	11	4.4%	-0.55 [-1.38, 0.29]
Pang, RY, et al 2021 A	10.22	1.46	3	13.1	0.94	3	2.1%	-1.88 [-4.29, 0.54]
Ruan J, et al 2014 A	9.01	0.52	9	11.37	0.3	9	2.4%	-5.29 [-7.46, -3.13]
Ruan J, et al 2014 B	8.85	0.35	9	11.48	0.74	9	2.8%	-4.33 [-6.17, -2.48]
Xue M, et al 2018	8	1.8	15	8.4	2.1	15	4.6%	-0.20 [-0.92, 0.52]
Zhu JJ, et al 2014 A	4.9	1.1	5	6.3	0.8	5	3.4%	-1.31 [-2.76, 0.13]
Zhu JJ, et al 2014 B	5.5	0.9	5	6.2	0.9	5	3.6%	-0.70 [-2.00, 0.60]
Zhu JJ, et al 2014 C	5.3	1.4	5	6.6	0.5	5	3.5%	-1.12 [-2.51, 0.27]
**Subtotal (95% CI)**			**103**			**102**	**42.8%**	**-1.48 [-2.27, -0.69]**
Heterogeneity: Tau² = 1.45; Chi² = 56.00, df = 11 (P < 0.00001); I² = 80%								
Test for overall effect: Z = 3.69 (P = 0.0002)								
**2.3.3 200＜TP≤400ug/(Kg·d)**								
Pang, RY, et al 2021 B	9.4	1.54	3	13.1	0.94	3	1.8%	-2.32 [-5.07, 0.43]
Pang, RY, et al 2021 C	8.45	1.62	3	13.1	0.94	3	1.5%	-2.81 [-5.96, 0.34]
Zhu JJ, et al 2014 D	4.9	1.1	5	6.3	0.8	5	3.4%	-1.31 [-2.76, 0.13]
Zhu JJ, et al 2014 E	5.7	0.8	5	6.2	0.9	5	3.7%	-0.53 [-1.81, 0.74]
Zhu JJ, et al 2014 F	5.5	0.8	5	6.6	0.5	5	3.3%	-1.49 [-2.99, 0.01]
**Subtotal (95% CI)**			**21**			**21**	**13.7%**	**-1.25 [-2.00, -0.50]**
Heterogeneity: Tau² = 0.00; Chi² = 2.85, df = 4 (P = 0.58); I² = 0%								
Test for overall effect: Z = 3.25 (P = 0.001)								
**2.3.4 TP＞400ug/(Kg·d)**								
Guo H， et al 2016 A	9.06	1.17	15	9.14	0.74	15	4.6%	-0.08 [-0.80, 0.64]
Guo H， et al 2016 B	9.83	0.89	15	9.14	0.74	15	4.6%	0.82 [0.07, 1.57]
Guo H， et al 2016 C	9.95	1.13	15	9.14	0.74	15	4.6%	0.83 [0.08, 1.58]
**Subtotal (95% CI)**			**45**			**45**	**13.7%**	**0.51 [-0.09, 1.11]**
Heterogeneity: Tau² = 0.14; Chi² = 3.94, df = 2 (P = 0.14); I² = 49%								
Test for overall effect: Z = 1.68 (P = 0.09)								
**Total (95% CI)**			**233**			**231**	**100.0%**	**-0.93 [-1.38, -0.47]**
Heterogeneity: Tau² = 1.03; Chi² = 116.00, df = 27 (P < 0.00001); I² = 77%								
Test for overall effect: Z = 4.00 (P < 0.0001)								
Test for subgroup differences: Chi² = 20.78, df = 3 (P = 0.0001), I² = 85.6%								

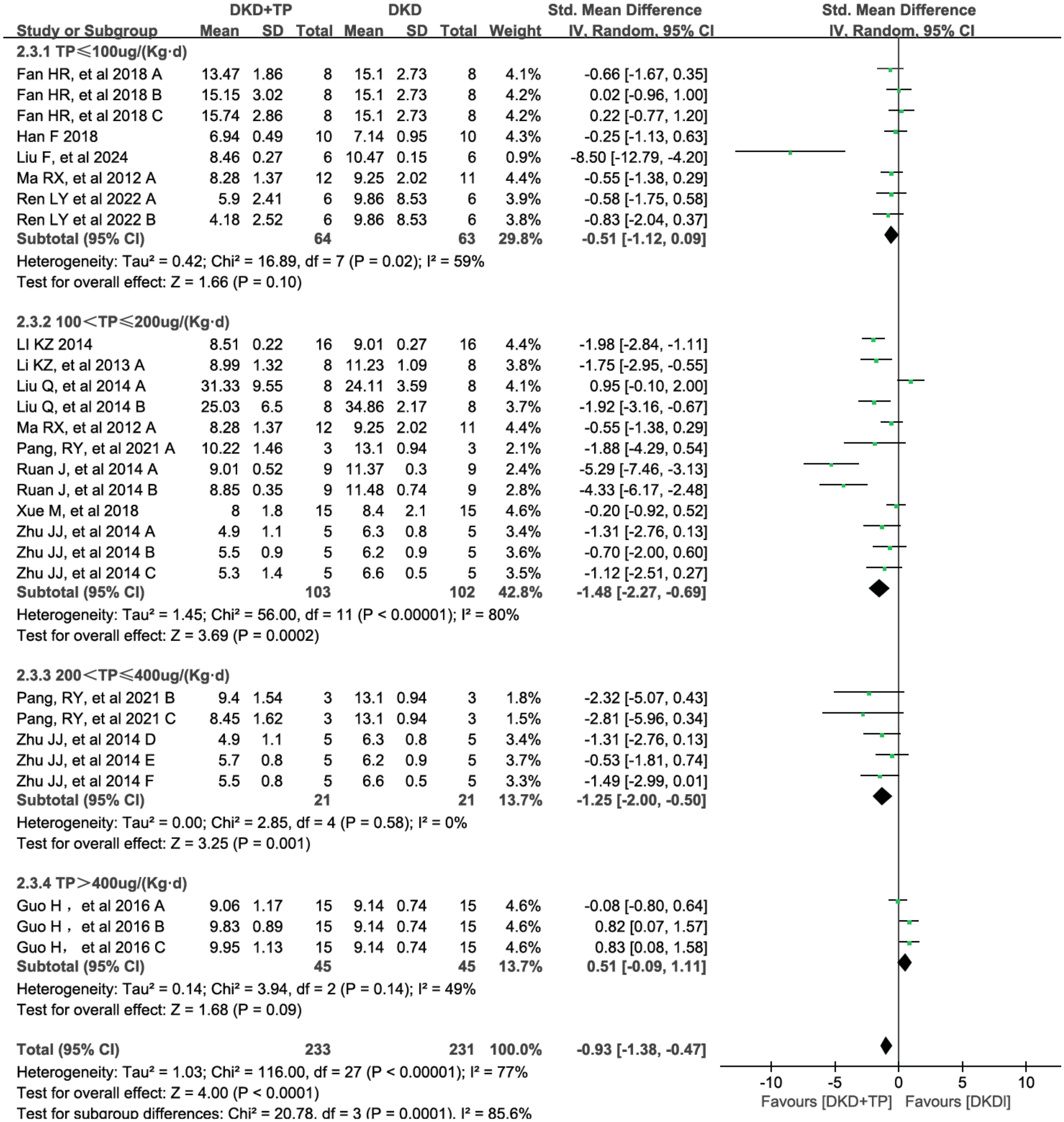

Fig. (S3). Fig. Subgroup analysis of the effect of different doses of TP on BUN in DKD.
